# Controls on δ^26^Mg variability in three Central European headwater catchments characterized by contrasting bedrock chemistry and contrasting inputs of atmospheric pollutants

**DOI:** 10.1371/journal.pone.0242915

**Published:** 2020-11-30

**Authors:** Martin Novak, Juraj Farkas, Pavel Kram, Jakub Hruska, Marketa Stepanova, Frantisek Veselovsky, Jan Curik, Alexandre V. Andronikov, Ondrej Sebek, Martin Simecek, Daniela Fottova, Leona Bohdalkova, Eva Prechova, Magdalena Koubova, Hyacinta Vitkova

**Affiliations:** 1 Czech Geological Survey, Prague 5, Czech Republic; 2 Department of Earth Sciences, Metal Isotope Group (MIG), The University of Adelaide, North Terrace, Adelaide, Australia; Trent University, CANADA

## Abstract

Magnesium isotope ratios (^26^Mg/^24^Mg) can provide insights into the origin of Mg pools and fluxes in catchments where Mg sources have distinct isotope compositions, and the direction and magnitude of Mg isotope fractionations are known. Variability in Mg isotope compositions was investigated in three small, spruce-forested catchments in the Czech Republic (Central Europe) situated along an industrial pollution gradient. The following combinations of catchment characteristics were selected for the study: low-Mg bedrock + low Mg deposition (site LYS, underlain by leucogranite); high-Mg bedrock + low Mg deposition (site PLB, underlain by serpentinite), and low-Mg bedrock + high Mg deposition (site UDL, underlain by orthogneiss). UDL, affected by spruce die-back due to acid rain, was the only investigated site where dolomite was applied to mitigate forest decline. The δ^26^Mg values of 10 catchment compartments were determined on pooled subsamples. At LYS, a wide range of δ^26^Mg values was observed across the compartments, from -3.38 ‰ (bedrock) to -2.88 ‰ (soil), -1.48% (open-area precipitation), -1.34 ‰ (throughfall), -1.19 ‰ (soil water), -0.99 ‰ (xylem), -0.95 ‰ (needles), -0.82 ‰ (bark), -0.76 ‰ (fine roots), and -0.76 ‰ (runoff). The δ^26^Mg values at UDL spanned 1.32 ‰ and were thus less variable, compared to LYS. Magnesium at PLB was isotopically relatively homogeneous. The δ^26^Mg systematics was consistent with geogenic control of runoff Mg at PLB. Mainly atmospheric/biological control of runoff Mg was indicated at UDL, and possibly also at LYS. Our sites did not exhibit the combination of low-δ^26^Mg runoff and high-δ^26^Mg weathering products (secondary clay minerals) reported from several previously studied sites. Six years after the end of liming at UDL, Mg derived from dolomite was isotopically undetectable in runoff.

## Introduction

Three to four decades after the reversal of acidification in industrial countries of Europe and North America, healthy young conifer and broadleaf stands are reported from spruce die-back affected areas [1, 2]. High atmospheric inputs of strong acids (H_2_SO_4_, HNO_3_) in the years of peak pollution (1975–1995) intensified leaching of bioavailable calcium (Ca) and magnesium (Mg) from the soils and their export from headwater catchments *via* surface runoff [3–8]. The provenance of Ca and Mg in present-day runoff from small forested catchments can be inferred from predictive hydrochemical models [9–11]. However, empirical evidence based on isotope studies or field-scale manipulations is scarce. Here we report Mg isotope systematics in three forested, high-elevation catchments in the Czech Republic (Central Europe) affected by acid rain. In the so-called Black Triangle (northern Czech Republic, southeastern Germany and southwestern Poland), spruce died back on the territory of 1000 km^2^ [12–14]. Since the 1990s, the growth rate of Norway spruce [*Picea abies*] throughout the Czech Republic has accelerated, indicating lower SO_2_ emissions, lower Al toxicity, and an increasing supply of nutrients [15].

Magnesium serves as the coordinating cation in the molecule of chlorophyll and activates enzymes needed for the synthesis of organic compounds [16]. Data on the isotope composition of Mg (^26^Mg/^24^Mg ratios, expressed in δ^26^Mg values) can provide new insights into the biogeochemical cycling of Mg. Studies focusing on the behavior of Mg isotope fractionations in terrestrial ecosystems have been reviewed by [17–20]. On crystalline bedrock, much of the within-site variability in δ^26^Mg values appears to be driven by inorganic isotope fractionations accompanying the conversion of primary silicates to secondary clay minerals [17]. Silicate weathering typically results in low δ^26^Mg values of runoff, and high δ^26^Mg values in clay minerals [21, 22]. Recent studies, however, have shown that the direction of Mg fractionation during weathering can be mineral-specific [23–28]. Also soil exchange processes can be isotope-selective [29]. Biological Mg isotope fractionations result in both lower and higher δ^26^Mg values of organic compounds relative to the source of bioavailable Mg [30–39]. One prerequisite of a combined isotope and mass-balance approach to Mg source apportionment in small catchments is that the hydrological conditions are well understood, and that the subsurface export of Mg *via* trans-regional groundwater is insignificant. Another assumption for a successful use of Mg isotope ratios for the determination of the origin of various ecosystem Mg compartments and fluxes is that the Mg sources are isotopically distinct and that direction and magnitude of Mg isotope fractionations are well understood.

Comparisons between catchments located along Mg availability gradients are an under-exploited tool in biogeochemical studies. For the present study, we selected three small headwater catchments with contrasting Mg availability: (i) low-Mg bedrock (leucogranite) + low Mg deposition; (ii) high-Mg bedrock (serpentinite) + low Mg deposition, and (iii) low-Mg bedrock (orthogneiss) + high Mg deposition. The high atmospheric deposition of Mg-rich dust at the third study site originated from a cluster of coal-fired power plants near the Czech-Polish border. Using Mg isotope data from 10 ecosystem compartments, our objectives were: (i) to quantify the catchment-scale δ^26^Mg variability along the gradient of ecosystem Mg availability; and (ii) to constrain the origin of Mg in catchment runoff among the contrasting study sites. Additionally, we combined the Mg isotope study with long-term monitoring of hydrochemical Mg mass budgets and Mg inventories. Hydrochemical data were used to evaluate whether the studied catchments were a sink or a source of Mg. We hypothesized that δ^26^Mg values of runoff in the studied industrial part of Central Europe do not reflect only lithogenic and biological controls, but are also consistent with the presence of recent atmospheric deposition in catchment runoff.

## Methods

### Study sites

**Fig 1:**
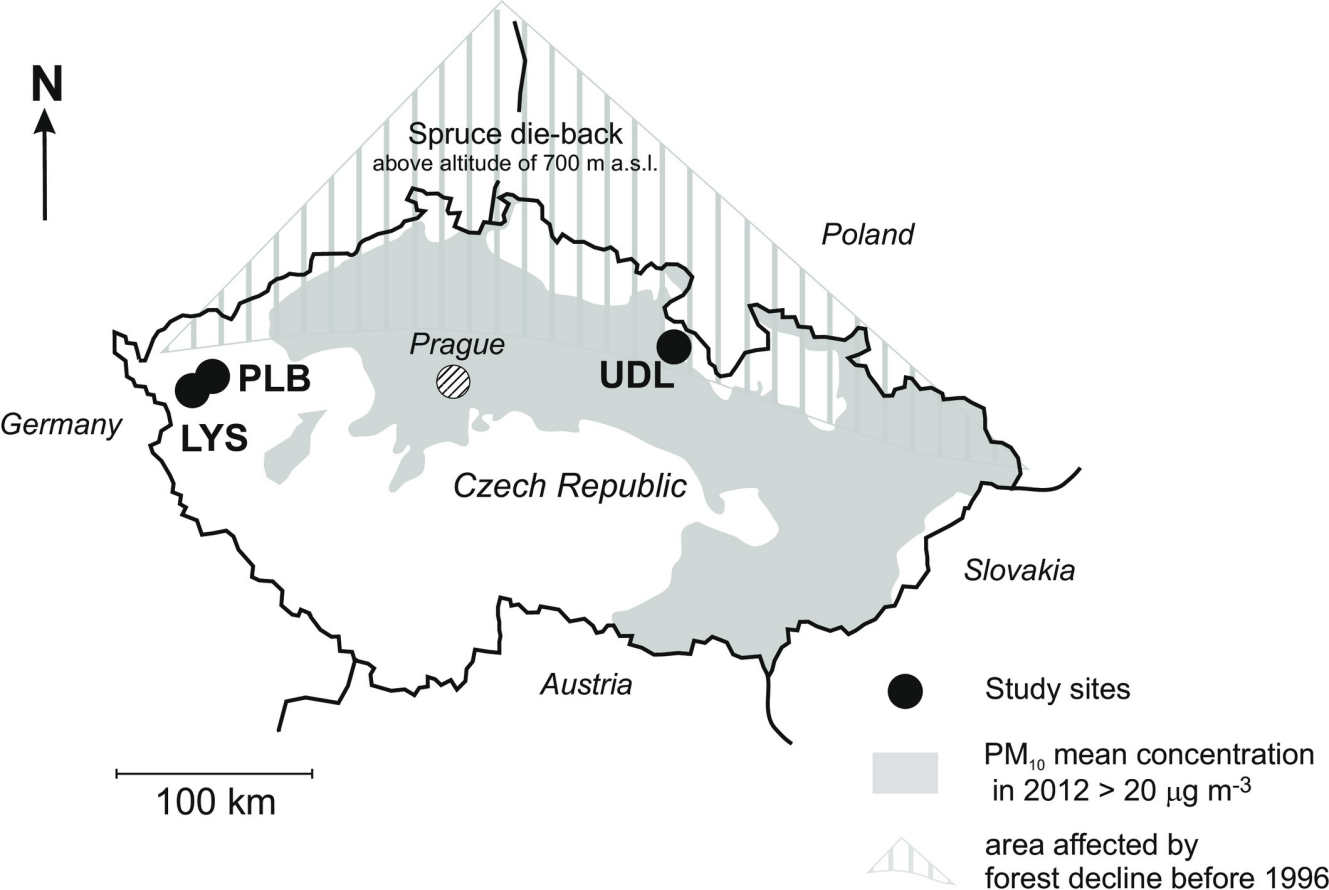
Location of the study sites. Data on particulate matter (PM10) are by Czech Hydrometeorological Institute, Prague (*portal*.*chmi*.*cz;* public domain data).

The three small headwater catchments included in our Mg-isotope study (Fig 1) are part of the GEOMON hydrochemical monitoring network (Czech Republic; [40, 41]). Sites for the current study were selected to capture extremes in Mg availability: Lysina (LYS, Slavkov Forest, west Bohemia; [10, 41–44]) is characterized by low Mg inputs *via* bedrock dissolution through chemical weathering, and relatively low Mg inputs *via* atmospheric deposition of pollutants. The LYS bedrock is leucogranite (0.15 wt. % of MgO; S1 Table). Pluhuv Bor (PLB; Slavkov Forest, west Bohemia; [10, 41–45]) is characterized by high Mg inputs *via* bedrock dissolution, and relatively low Mg inputs *via* atmospheric deposition. The PLB bedrock is mainly serpentinite (36.0 wt. % of MgO; S1 Table). The distance between LYS and PLB is seven km. U Dvou Loucek (UDL; Eagle Mts., northeastern Bohemia [41, 44, 46, 47]) is characterized by low Mg inputs *via* bedrock dissolution, and high Mg inputs *via* atmospheric deposition. The UDL bedrock is formed by orthogneiss (0.36 wt. % of MgO; S1 Table). The distance of UDL from LYS and PLB is approximately 230 km. Due to acid rain, UDL suffered massive Norway spruce die-back in the 1980s and 1990s. The UDL catchment was treated with dolomite [(CaMg(CO_3_)_2_] to mitigate the impact of acidic deposition. A total of nine tons of dolomitic limestone per hectare were applied between 1980 and 2007. Climatic conditions at the study sites and soil characteristics are given in Table 1. The field site access was approved by the land owner [Lesy CR (LYS and PLB), and the Kolowrat Estate (UDL)].

**Table 1:** Study site characteristics.

Site	Lysina, Slavkov Forest	U Dvou Loucek, Eagle Mountains	Pluhuv Bor, Slavkov Forest
Acronym	LYS	UDL	PLB
Location	50^o^03´ N 12^o^40´ E	50^o^13´ N 16^o^29´ E	50^o^04´ N 12^o^46´ E
Catchment area (ha)	27	33	22
Elevation (m)	829–949	880–950	690–804
Aspect	North-east	South-west	South-east
Bedrock	Leucogranite	Orthogneiss	Serpentinite
Soil type	Podzol, Cambisol	Spodo-dystrict cambisol, Cambic podzol	Magnesic cambisol, Stagnosol
Mean annual precipitation (mm)	990	1310	800
Snow cover (days)	90	100	70
Mean winter temperature (°C)	-5.0	-5.0	-4.0
Mean summer temperature (°C)	14	15	15
Vegetation	Norway spruce (*Picea abies*), *Calamagrostis villosa*	Norway spruce (*Picea abies*), European beech (*Fagus sylvatica*), Fir (*Abies alba)*, *Calamagrostis villosa*, *Deschampsia*, *Oxalis*	Norway spruce (*Picea abies*), Scots pine (*Pinus sylvestris)*
Typical forest age (years)*	41–60	21–40	61–100
Mature spruce forest area (ha) *	27	3	18
Young (<40 years) spruce forest (ha) *	0	27	0
Broadleaf forest area (ha) *	0	1.5	0
Unforested area (ha)*	0.4	1.7	4.0

*data for 2015

### Sampling

#### Hydrochemical input–output monitoring

The long-term hydrochemical monitoring at LYS, UDL and PLB included two types of atmospheric inputs, open-area precipitation and spruce canopy throughfall, and catchment output *via* streamwater. Here we report a time-series of Mg fluxes for the hydrological years 1994–2012.

Two samplers installed five meters apart were used to collect open-area precipitation. For the collection of spruce throughfall, arrays of five samplers installed in two plots were used at LYS and PLB. At UDL, an array of nine samplers in a 10 × 10 m grid was used. The samplers were polyethylene (PE) funnels (122 cm^2^) with a 0.05 mm mesh fitted to PE 1-L bottles placed 120 cm above the ground. Snow was sampled using cylindrical plastic collectors, 50 cm tall (167 cm^2^). Cumulative samples of rainfall and throughfall represented the entire month. At each site, a 1-L runoff sample was collected once a month near the gauging station.

#### Mg isotope study

Water samples for Mg isotope measurements were collected in May 2012 at LYS and PLB, and in January, April, July and October 2013 at UDL. The Mg isotope determinations were thus performed at the end of the 19-year monitoring period and six years after the last application of dolomitic limestone at UDL. Soil water at UDL was collected by suction lysimeters from the depths of 30 and 50 cm [47]. At LYS and PLB, gravitational lysimeters installed at depths of 10 and 20 cm were complemented with suction lysimeters at the depths of 60 and 90 cm. Cumulative monthly samples of soil water from three replicate lysimeters at each depth were pooled. All water samples for Mg isotope analysis were filtered. At UDL, nested suction lysimeters were sampled along a V-shaped valley (three sites differing in elevation on each slope of the valley; *see* [44] for details). Solid samples for the Mg isotope study in each catchment included: bedrock, soil, fine roots, xylem, needles from the last growing season, and bark of Norway spruce. Ten grab samples of bedrock fragments were collected throughout each catchment and pooled, while soil samples were collected from 2–3 depth intervals in three different soil pits and pooled within each depth interval. Spruce needles, fine roots and bark were collected from 10 trees throughout each catchment and pooled. Using an increment borer, xylem cores were collected at breast height (1.3 m) from three spruce stems at each site and pooled. Fine roots were washed thoroughly by deionized water to remove soil particles. The mass of each combined sample of bedrock fragments and soil depth interval was 2 and 1 kg, respectively, the dry mass of organic materials was approximately 0.8 kg, with the exception of xylem (0.15 kg). The bedrock samples were crushed after the weathering rinds were removed, and milled to < 0.64 mm. The δ^26^Mg values were determined also on dolomite collected from a temporary storage site in the vicinity of UDL in 2013.

The sampling design chosen for our reconnaissance study is, to some extent, capable of capturing Mg isotope differences among individual sample types, but not δ^26^Mg variability within a single sample type.

Isotope composition of atmospherically deposited elements in industrial areas can, in principle, differ along the catchment slope. We have previously documented spatial gradients in isotope composition of sulfur (S) in a Czech catchment characterized by a 500 m elevation span, and located mere 4 km from a coal-burning power plant [48]. The sites in the current Mg isotope study, however, are characterized by less steep slopes (elevation span of 70–120 m; Table 1), and their distance from point sources of industrial pollution are > 20 km. Therefore, we decided to sample only one research plot in our Mg isotope study.

#### Mg pool size inventory

Following field sampling in the summer of 2015, Mg pool sizes were calculated for aboveground and belowground vegetation (spruce trees), spruce needles, forest floor, and four soil depth intervals (0–10, 10–20, 20–40, 40–80 cm) at each site. Seven to nine quantitative soil pits per site used a 0.5 m^2^ reference frame. The excavated material was weighed and sieved through a 1-cm sieve, separated into stones, the < 1-cm soil fraction and coarse roots. Two kg of sieved soil were taken to the laboratory where moisture was determined after 24 hours of oven drying at 105°C. The bulk soil samples were air-dried and sieved to obtain a < 2 mm fraction for chemical analysis.

### Analysis

Magnesium concentrations in water were determined by flame absorption atomic spectrometry (FAAS; limit of determination 0.01 mg L^-1^). Water runoff fluxes were determined using continuous data from gauging stations in each catchment [40, 41]. Annual Mg fluxes were calculated from the measured concentrations and water fluxes. Magnesium budgets for each catchment in kg ha^-1^ yr^-1^ were calculated as a difference of atmospheric inputs minus runoff outputs.

Magnesium isotope analyses were carried out in the laboratory of the Czech Geological Survey, Prague, using the methodology of Shalev et al. [49] and ultrapure reagents. Carbonate powders for Mg isotope analysis were digested in 6 M HCl. Homogenized bedrock and soil samples were digested in a 1:1 mixture of concentrated HF and HNO_3_ to break down the silicate fraction; a follow-up digest in concentrated HCl was performed if insoluble fluorides were present. Homogenized organic-rich materials were digested in a 1:1 mixture of concentrated H_2_O_2_ and HNO_3_ to break down organic compounds. Aliquots of water samples containing approximately 20 μg Mg were evaporated to dryness and treated with 1 mL of concentrated HNO_3_. Solutions with visible solid residues were re-dissolved in a 1:1 mixture of concentrated H_2_O_2_ and HNO_3_. Samples were then evaporated to dryness and re-dissolved in 3% v/v HNO_3_. Prior to loading onto columns, the Mg samples were again evaporated to dryness and re-dissolved in 100 μL of 2.5 M HCl. Magnesium purification was accomplished by a three-step chromatographic separation using 1.25 mL of the Bio-Rad AG 50W-X12, 200–400 mesh resin in Savillex PFA 3.2 x 20 cm microcolumns (steps no. 1 and 3), and 0.12 mL resin in 2.4 mm x 15 cm microcolumns (step no. 2). In the first step, the Mg fraction which still contained Na and Fe, was separated from other matrix elements by elution with 4.9 M of 2.5 M HCl. In the second step, Mg with Fe was separated from Na by elution with 4.9 mL of 0.4 M HCl and collected with 1.5 mL of 6 M HCl. In the third step, the Mg fraction was separated from Fe by rinsing with 5.2 mL of 2 M HNO_3_ prior to elution of the purified Mg with 5.5 mL of 2 M HNO_3_. The purified Mg fraction was then evaporated to dryness, re-dissolved in a 200 μL of concentrated 1:1 mixture of HF and HNO_3_, evaporated to dryness again, re-dissolved in a concentrated 1:1 H_2_O_2_-HNO_3_ mixture, evaporated to dryness and finally dissolved in 3% v/v HNO_3_. The Mg recovery was complete (100%), based on the calibration of the chemical purification procedure and recovery yields for Mg, for details see [49]. The total procedural blank was < 3 ng Mg, which was significantly lower (< 0.01%) than the total amount of Mg (typically 20 μg) originating from a sample processed through column chemistry and analyzed for the Mg isotope composition. Magnesium isotope ratio measurements were performed on a Thermo Scientific *Neptune* MC-ICP-MS, equipped with a Ni sampler and X-Version Ni skimmer cones. Solutions containing 500 ng Mg mL^-1^ were introduced into the plasma *via* a 100 μL min^-1^ PFA nebulizer and a cyclonic quartz-glass spray chamber. The following cup configuration was used: the ion beam intensities at m/z 24, 24 and 26 were measured simultaneously using Faraday cups L1, C and H2. All measurements were carried out in a medium mass-resolution mode. Typical signals on m/z 24 were 4 to 8 V. Each measurement comprised 30 cycles with an 8.4 s acquisition time. Isotope composition of Mg is reported in δ^26^Mg values as per mil (‰) deviation of the ^26^Mg/^24^Mg ratio of a sample relative to the ^26^Mg/^24^Mg ratio of a bracketing standard:

$$\delta^{26}\mathrm{Mg}\left(‰\right)=\left[\left({}^{26}\mathrm{Mg}{/}^{24}{\mathrm{Mg}}_{\mathrm{sample}}\right)/\left({}^{26}\mathrm{Mg}{/}^{24}{\mathrm{Mg}}_{\mathrm{DSM}\;\mathrm{standard}}\right)-1\right]\;x\;1000$$

Each single δ value was calculated by at least three repeated measurements of the same sample solution, each bracketed by DSM3 (typical reproducibility of δ^26^Mg of -0.00 ± 0.08 ‰ n = 3). Procedural blank contributions including background were consistently below 0.1% of sample signals and therefore no on-peak blank corrections were applied. Electronic background was collected at half mass unit before each block and subtracted from the measured signals. The acquired external reproducibility and precision of the δ^26^Mg and δ^25^Mg values for the DSM3 standard was ± 0.13 ‰ and ± 0.09 ‰, respectively (2σ; n = 20). The external precision was established by repeated analysis of NIST SRM 1515 (δ^26^Mg of -1.24 ± 0.08 ‰), JDo-1 (-2.49 ± 0.03 ‰), and IAPSO Atlantic Seawater (OSIL; -0.87 ± 0.03 ‰, *cf*., [50]). For δ^26^Mg results of repeated analysis of another six internationally established Mg isotope reference materials (BCS-CRM 512, Cambridge-1, DSW-1, SLRS-5, NISR SRM 1640a, and NIST SRM 2709a) *see* [49]). For the linear relationship between δ^25^Mg and δ^26^Mg ratios of the DSM3 standard also *see* [49].

Concentrations of exchangeable Mg for the catchment-level Mg inventory were determined in BaCl_2_ soil extracts. A 2.5 g soil sample (< 2 mm) was mixed with 30 mL of 0.1 M BaCl_2_, shaken for 2 hours, centrifuged for 10 minutes, and Mg concentrations were determined using FAAS. For each 400-m^2^ plot, dry biomass samples (needles, xylem, fine roots) were combusted at 550°C and dissolved in concentrated HF/saturated H_3_BO_3_. After evaporation, the samples were dissolved in HCl, concentrations of Mg were determined using AAS. Biomass pools in 2015 were calculated using general allometric equations and site-specific diameters at breast height (DBH).

We assumed that Mg isotope analysis of exchangeable Mg would give similar results as Mg isotope analysis of soil water collected by lysimeters and therefore measured δ^26^Mg values of soil water only (see [32] for a detailed discussion of the topic).

X-ray diffraction analysis (XRD) was used to identify clay minerals at LYS, UDL and PLB in three replicated samples of mineral soil from the depth of 40–80 cm. Powder mounts were prepared from ground samples of the < 2 mm soil fraction by back-side filling. Fifteen wt. % of ZnO were added as an internal standard. The <2 μm fraction was separated by a sedimentation technique in distilled water [51]. K-saturation of the separated clay fraction was reached by five saturation cycles with a 1M KCl solution, followed by washing of the suspension in distilled water to a negative reaction with AgNO_3_. Oriented preparations of the clay fraction, obtained by 2.5 mL of suspension pipetting on the Si-slide, were exposed to ethylene-glycol vapour at 60°C for 8 h. Oriented preparations were heated to 110°C for 5 h, and to 300°C for 2h. XRD analyses were performed with a Bruker D8 Advance Diffractometer (CuKα, primary and secondary Soller slits 2.5°, detector Lynxeye XE) with an automatic divergence slit (ADS, 10 mm).

Back-side loaded, random powder preparations of milled bulk soil samples were recorded within the range of 4–80°2Θ with a step of 0.015°2Θ, and time per step of 0.8 s. The mineral phases from bulk soil samples were identified from XRD patterns by the ZDS-WX software [52] and the PDF-2 database [53]. Semiquantitative phase analyses were performed using the Rietveld method by Topas 5 software [54]. Models of crystal structures data of identified minerals from the ICSD database [55] were used. The content of mixed-layered minerals was estimated from the determined amorphous material. X-ray diffraction patterns of oriented preparations were recorded within the range of 2.8–50°2Θ with a step of 0.019°2Θ, and time per step of 0.8 s. Interstratified clay minerals were identified by comparison of the XRD patterns with patterns modelled by the NEWMOD software [56]. Mixed-layered minerals were identified according to [57].

The statistical analysis was performed using the R software [58], version 3.6.1. Input–output Mg fluxes were compared using the paired t-test. Pearson’s product moment correlation coefficients for individual types of liquid samples at UDL were determined along with one-sided P-values.

## Results

### Mg input–output fluxes

**Fig 2:**
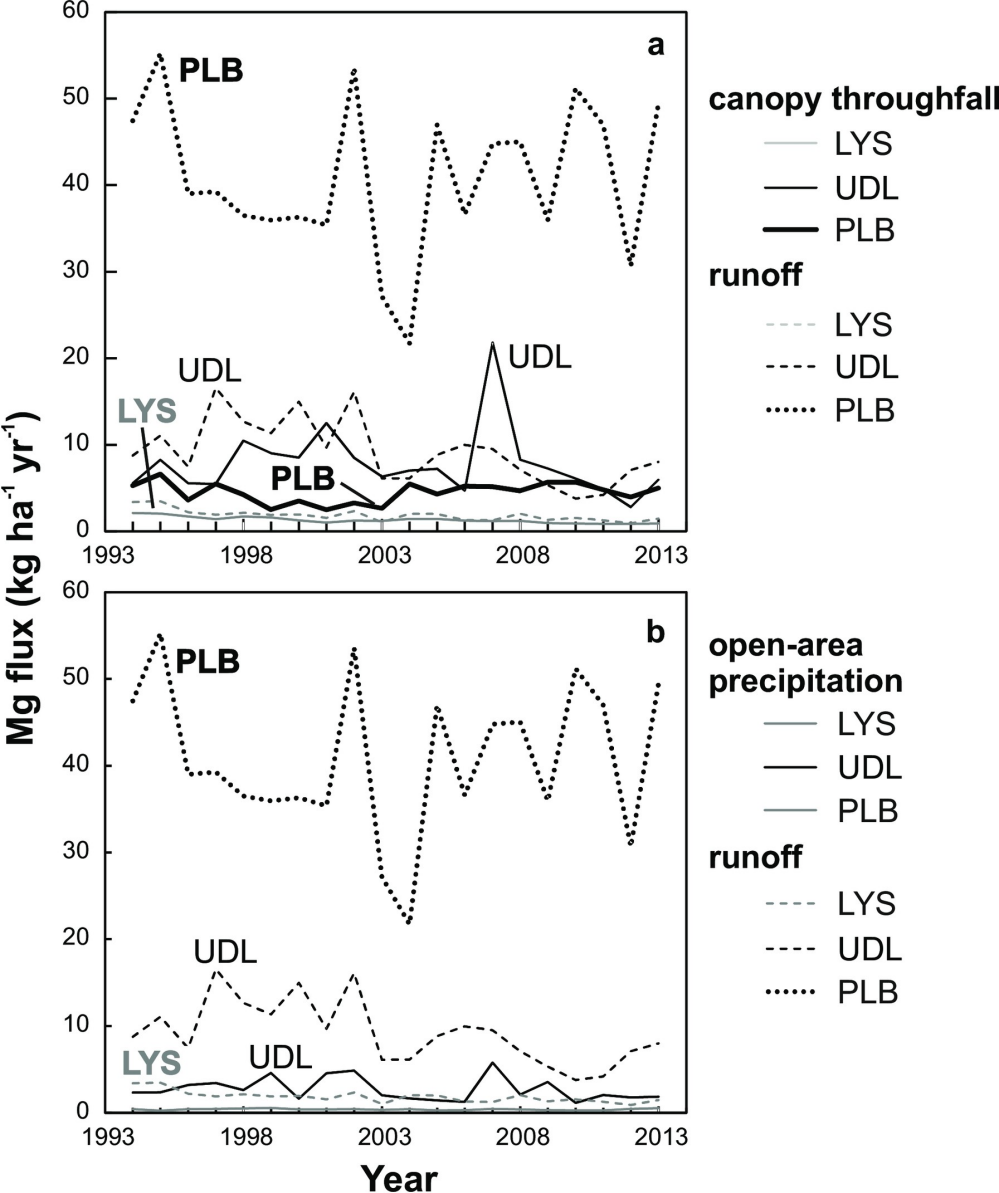
Time-series of annual Mg input–output fluxes.

Annual discharge-weighted mean runoff at Mg-rich PLB contained as much as 30 mg Mg L^-1^. Runoff from Mg-poor LYS and UDL was characterized by less than 0.6 and 1.2 mg Mg L^-1^, respectively. At all study sites, Mg concentrations in open-area precipitation were lower, compared to throughfall, soil solutions and runoff. There were no distinct long-term trends in Mg input–output fluxes at the study sites (Fig 2). Annual Mg fluxes typically increased in the order: open-area precipitation < throughfall < runoff. Runoff Mg fluxes at PLB were on average 4.4 times higher than those at UDL, and 22 times higher than those at LYS (means of 41, 9.2, and 1.9 kg Mg ha^-1^ yr^-1^, respectively). Long-term Mg deposition fluxes *via* open-area precipitation were low at LYS and PLB (0.4 to 0.5 kg ha^-1^ yr^-1^), and about five times higher at UDL (2.7 kg ha^-1^ yr^-1^). Mean Mg deposition *via* throughfall was 1.3 kg ha^-1^ yr^-1^ at LYS, *i*.*e*., 3.3 times higher than local Mg flux *via* open-area precipitation. Mean Mg flux *via* throughfall was 3.4 times higher at PLB than at LYS (4.5 *vs*. 1.3 kg ha^-1^ yr^-1^), and 5.9 times higher at UDL than at LYS (7.8 *vs*. 1.3 kg ha^-1^ yr^-1^). At UDL, Mg fluxes *via* throughfall and runoff between 1994 and 2012 were statistically indistinguishable (*p* = 0.199; Fig 2A).

### Mg pool sizes

The total Mg pools in the spruce trees (aboveground vegetation) increased in the order: LYS (23 kg ha^-1^) < UDL (42 kg ha^-1^) < PLB (47 kg ha^-1^ S1 Fig in the Electronic Annex). The total Mg pools in spruce roots (belowground vegetation) were 4 to 7 times lower compared to the aboveground total Mg pools. The exchangeable Mg pool size in the forest floor increased from LYS (5.9 kg ha^-1^), to UDL (32 kg ha^-1^), and to PLB (200 kg ha^-1^). The exchangeable Mg pool size in the 20–40 cm soil depth increased also from LYS (16 kg ha^-1^), to UDL (50 kg ha^-1^), and to PLB (1270 kg ha^-1^). At these soil depths, the most Mg-enriched site PLB contained more than 70 times more exchangeable Mg than the most Mg-depleted site LYS.

### Mg isotope systematics

**Fig 3:**
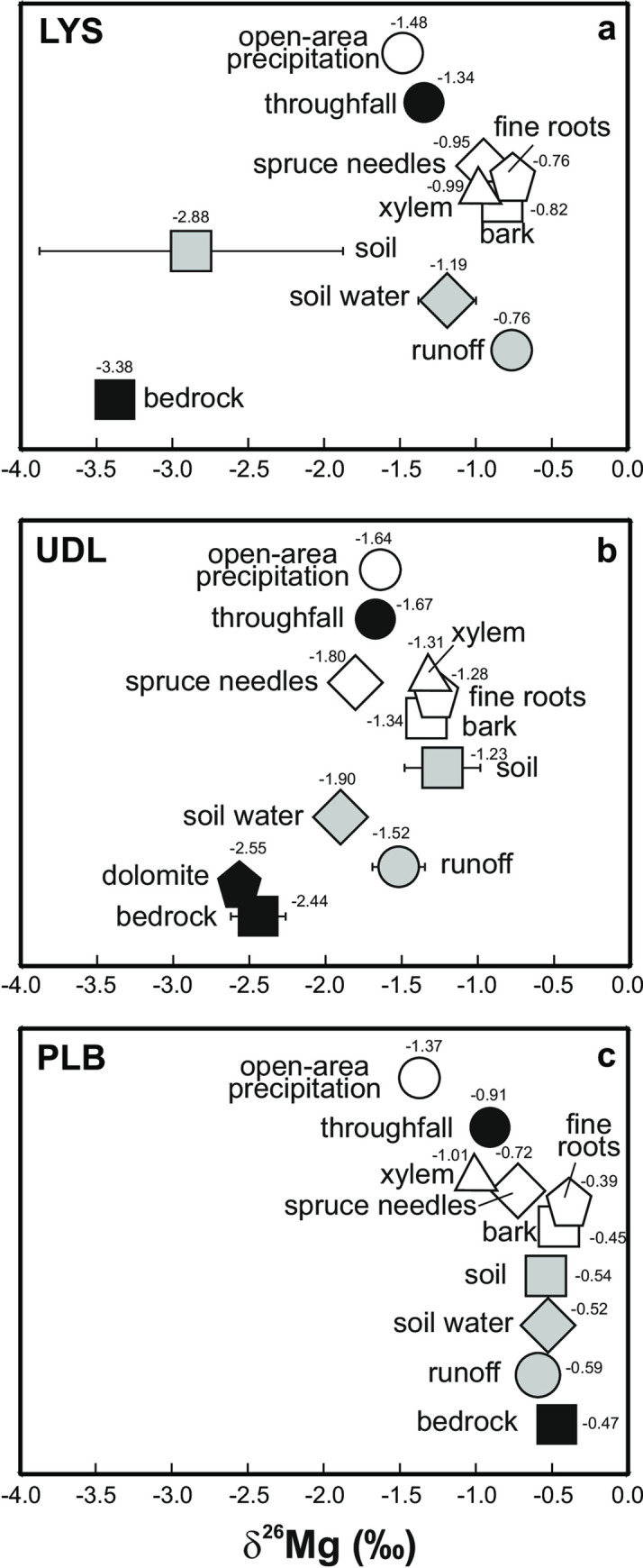
The measured Mg isotope systematics at LYS (a), UDL (b) and PLB (c). Arithmetic means are given for δ^26^Mg values from bulk soil profiles. For individual Mg isotope measurements *see* S2 Table.

In this paragraph, we focus on differences among the δ^26^Mg values of catchment compartments that were larger than the reproducibility of mass spectrometric measurements (0.26 ‰). At LYS and UDL, local weathered bedrock was characterized by the lowest δ^26^Mg values among all compartments (-3.38 and -2.55 ‰, respectively; Fig 3A and 3B, and S2 Table). At LYS, Mg in the analyzed samples of runoff had higher δ^26^Mg values not only compared to bedrock, but also relative to the analyzed samples of atmospheric deposition (δ^26^Mg value of runoff and spruce canopy throughfall were -0.76 and -1.34 ‰; Fig 3A and 3B, and S2 Table). At both of these sites, the measured δ^26^Mg values of individual *Picea abies* tissue types clustered around the δ^26^Mg value of runoff; the studied organic Mg pools at UDL had higher δ^26^Mg values, compared to soil water. Dolomite applied on the surface of UDL contained low-δ^26^Mg magnesium (-2.55 ‰), similar to bedrock orthogneiss (-2.44 ‰). Bulk soil represented an exception from the Mg isotope similarity between LYS and UDL. Magnesium in bulk soil at LYS was characterized by low δ^26^Mg values (mean of -2.88 ‰), whereas Mg in bulk soil at UDL had the highest δ^26^Mg values (mean of -1.23 ‰).

At PLB, the overall range of δ^26^Mg signatures was narrower, compared to LYS and UDL (Fig 3C; S2 Table). At PLB, bedrock, soil, soil water and runoff had nearly identical δ^26^Mg values between -0.59 and -0.47 ‰. Individual *Picea abies* tissue types exhibited a broader range of δ^26^Mg values from -1.0 to -0.39 ‰. The measured δ^26^Mg value of xylem plotted between the δ^26^Mg values of open-area precipitation (-1.37 ‰) and throughfall (-0.91 ‰). Open-area precipitation was characterized by the lowest δ^26^Mg value among all PLB catchment compartments.

### Temporal trend in δ^26^Mg values

**Fig 4:**
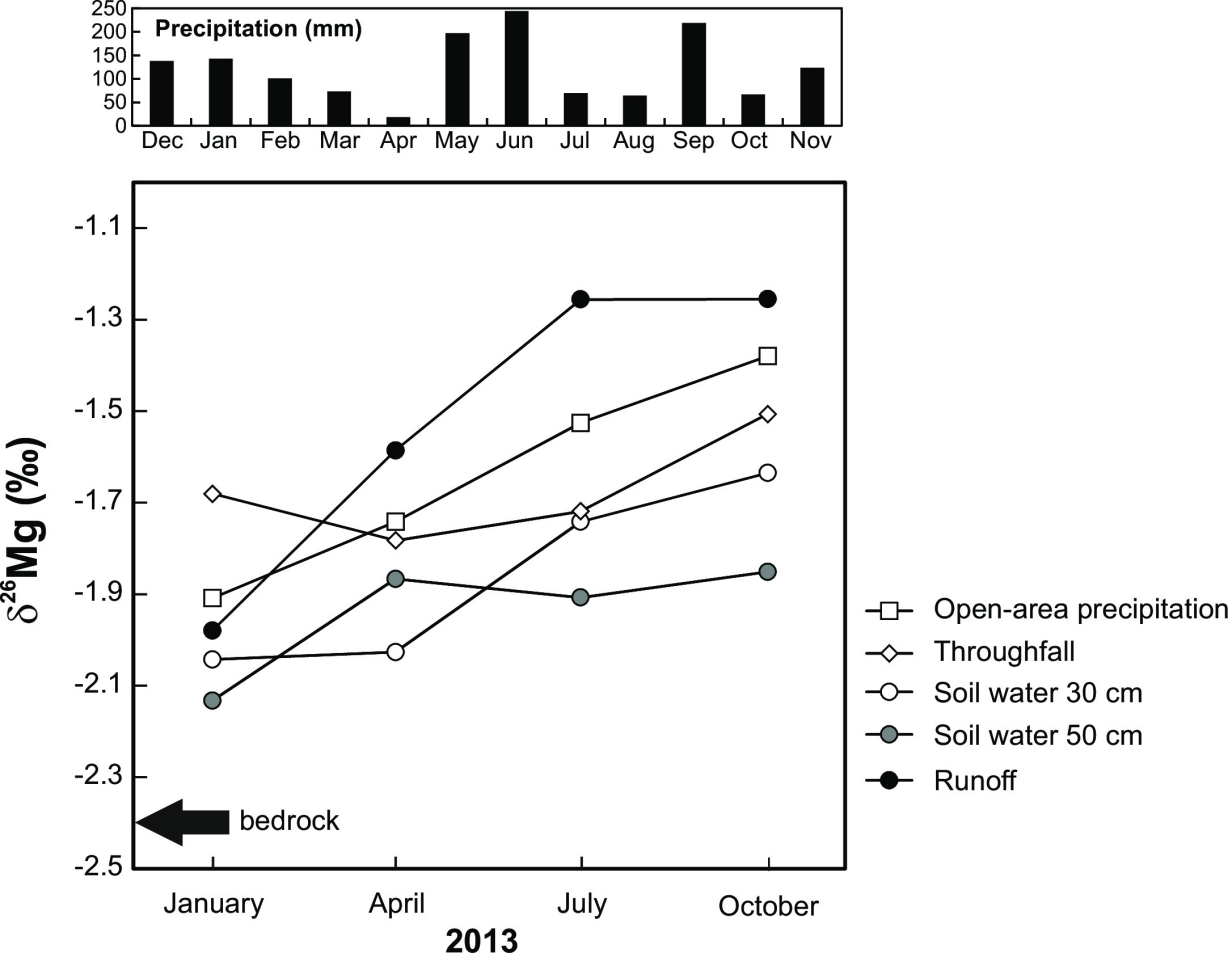
Temporal trend in δ^26^Mg values of individual types of water samples at UDL. Magnesium concentrations in runoff roughly correspond to those of soil water; Mg in soil water is more concentrated than Mg in rainfall due to evapotranspiration, biological cycling, and water-rock interaction (Table 1).

At UDL, we determined δ^26^Mg values of atmospheric deposition (open-area precipitation and throughfall), soil water (two depths), and runoff in the spring, summer, fall, and winter of 2013. All five variables exhibited a general trend toward higher δ^26^Mg values from January to October 2013 (Fig 4). The positive correlation was statistically significant between open-area precipitation and soil water at 30-cm depth (*R* = 0.96, *p* = 0.018), and between open area-precipitation and runoff (*R* = 0.95, *p* = 0.024; S3 Table).

The lowest δ^26^Mg value of UDL runoff, the isotopically most variable water type, was 0.46 ‰ higher than the δ^26^Mg value of local bedrock. The lowest δ^26^Mg values of UDL open-area precipitation, throughfall, soil water at the 30-cm depth, and soil water at 50-cm depth were 0.53, 0.76, 0.40, and 0.31 ‰, respectively, higher than the δ^26^Mg value of bedrock (S2 Table). With respect to the reproducibility of the mass spectrometric measurements (± 0.13 ‰), these δ^26^Mg values of water samples were isotopically distinguishable from UDL bedrock.

### Newly-formed clay minerals in soil

X-ray diffraction identified only small amounts of kaolinite in the mineral soil (mean of 2.5 ± 0.5 wt. % at LYS, 2.3 ± 0.8 wt. % at UDL, and < 0.5 wt. % at PLB). No other clay minerals were detected.

## Discussion

### Comparison of trends in Mg fluxes with trends in S, N, and Ca fluxes

The lack of temporal trends in Mg input fluxes at our three study sites (Fig 2) contrasted with decreases in Ca deposition fluxes between 1994 and 2012 [44]. In contrast to Mg, decreases in Ca deposition fluxes reflected long-term decreases in the deposition of mostly acidifying sulfur (S) and reactive nitrogen (N), especially at the industrially most polluted site UDL [44]. Also temporal decreases in runoff fluxes of Ca were more distinct than those of Mg. In general, retreating acidification did not result in systematically decreased Mg fluxes, indicating at least partial de-coupling of Mg and Ca cycling. Magnesium deposition is, to a greater extent compared to Ca, affected by a contribution of sea-spray (*cf*., [41]).

### Sources of catchment-scale variability in δ^26^Mg values

#### PLB–ultramafic bedrock

The dissolution of Mg-rich serpentinite dominates Mg cycling at PLB (Fig 3C). The isotope composition of serpentinite is reflected in soil and soil water with no or negligible fractionation. The lack of Mg isotope fractionations during serpentinite weathering at PLB is interesting in light of previous reports that mineral dissolution is isotopically selective. Magnesium isotope fractionations may result in an enrichment in ^26^Mg in the weathering products (secondary clay minerals) and in a removal of dissolved ^24^Mg-rich magnesium *via* runoff [21, 25, 26, 29]. Soils at all sites in the current study are characterized by high Al-Fe sesquioxide content, with only small amounts of newly-formed clay minerals (< 3 wt. %). The erosion regime at these high-elevation sites does not generate conditions favorable for sizeable *in-situ* formation of clay minerals. Because Mg isotope ratios of bedrock and soil were nearly identical (Fig 3C), adsorption of Mg on Al-Fe sesquioxides did not result in a measurable isotope fractionation at PLB.

The analyzed xylem sample at PLB had lower δ^26^Mg values, compared to bedrock, soil and soil water (Fig 3C). The Mg isotope signature of PLB xylem was nearly identical with the Mg isotope signature of spruce throughfall, the predominating form of aboveground Mg input into the ecosystem. Yet, atmospheric deposition did not simply control the Mg isotope composition of xylem, with no isotope fractionation. As mentioned above, the higher δ^26^Mg values of the analyzed fine roots and needles compared to xylem provided evidence for the existence of biological Mg fractionation within the tree (*cf*., [37]). At PLB, lower biological Mg isotope fractionation accompanied higher exchangeable Mg content in the soil, compared to LYS and UDL (S1 Fig). Lower biological Mg isotope selectivity in an environment with high Mg availability is consistent with similar behavior of S, N, and Ca isotopes in ecosystems [59–62]. The magnitude of isotope fractionations of nutrients depends usually on the slowest step during assimilation; high nutrient availability may be associated with a relatively small isotope fractionation. We suggest that the Mg-rich bedrock at PLB, along with significantly higher Mg concentrations in bulk soil and soil water, compared to LYS and UDL (S2 Table), were likely responsible for the relatively small catchment-scale variability in δ^26^Mg values (Fig 3C). The δ^26^Mg variability at PLB was dominated by mixing of Mg deposited on the forest with geogenic Mg and a relatively small biological isotope effect.

#### LYS and UDL–felsic bedrock

The measured within-site ranges of δ^26^Mg values at LYS and UDL (2.62 and 1.21 ‰, respectively) were relatively large (Fig 3). Both LYS and UDL are similar to PLB in that *in-situ* formation of secondary clay minerals is limited (2.3 to 2.5 wt. % of soils). However, that does not necessarily imply that dissolution of felsic bedrock at these high-elevation sites and inorganic adsorption of geogenic Mg on Al-Fe sesquioxides in the soils did not fractionate Mg isotopes [*cf*., 39, 63]. The main rock-forming minerals in LYS leucogranite are orthoclase, albite, Li-mica, and quartz, while the main rock-forming minerals in UDL are orthoclase, albite, biotite, and quartz. Of these, only biotite (present at UDL in subordinated amounts) contains a large amount of Mg. In principle, we cannot rule out incongruent dissolution of minerals with contrasting δ^26^Mg values and inorganic Mg isotope fractionations during weathering as contributors to the observed difference between bedrock and soil.

The measured δ^26^Mg difference between bedrock and average soil at the two felsic sites was relatively large (0.50 ‰ at LYS, and 1.21 ‰ at UDL), which contrasted with PLB (0.07 ‰). At the felsic sites, Mg in both bulk soil and soil water was likely affected by biological Mg isotope fractionations. In general, within-site isotope-selective processes served as important controls of δ^26^Mg variability in felsic catchments characterized by contrasting atmospheric Mg deposition levels.

### Origin of Mg in catchment runoff

Oulehle et al. [41] summarized input–output mass balances of environmentally relevant elements in 15 headwater catchments of the Czech Republic. All those sites exported more Mg than they received *via* atmospheric deposition. Based on non-isotope data alone, it would seem likely that bedrock dissolution is responsible for the net export of Mg from the Czech headwater catchments, regardless of local lithology. This conclusion is clearly valid for PLB with as much as 36 wt. % of MgO in bedrock serpentinite (S1 Table), high Mg concentrations in soil solutions (up to 16 mg L^-1^), and nearly identical δ^26^Mg values of bedrock and runoff (Fig 3C). However, for the felsic sites LYS and UDL, the measured Mg isotope systematics alone (*i*.*e*., without considering input–output mass budgets), would indicate a large contribution of atmospherically deposited Mg to runoff, and a small contribution of geogenic Mg to runoff. The mean δ^26^Mg value of runoff at UDL was nearly the same as the mean δ^26^Mg value of atmospheric deposition, but different from the δ^26^Mg value of bedrock *and* the dolomite applied on the catchment surface (Fig 3B). The δ^26^Mg value of runoff at LYS was four times closer to the δ^26^Mg value of atmospheric deposition than to the δ^26^Mg value of bedrock; while the analyzed Mg in LYS runoff had a high δ^26^Mg value, Mg in LYS bedrock had a low δ^26^Mg value (Fig 3A). The question arises whether the mass-balance and isotope indicators of Mg origin in runoff from LYS and UDL can be reconciled.

By the year of our isotope study (2013), more than 80% of the surface of UDL was covered by a young spruce stand overgrowing clearings resulting from the forest decline of the 1980s (Table 1). Therefore, it is reasonable to consider the throughfall Mg flux (mean of 7.8 kg ha^-1^ yr^-1^) to be a better representation of catchment-level input of total dissolved Mg than the open-area deposition flux (mean of 2.7 kg ha^1^ yr^-1^; we need to keep in mind that some of the soluble Mg in throughfall was not *new* atmospheric Mg input, but rather resulted from biomass leaching, and ultimately originated from the soil). The throughfall Mg input flux at UDL was isotopically similar to open-area precipitation and to spruce needles (Fig 3B), and was thus consistent with two-way interaction between rainfall plus dry-deposited Mg and the needles with an only small Mg isotope fractionation accompanying chlorophyll formation. Importantly, the UDL time-series in Fig 2 yielded no statistically significant difference between Mg flux *via* spruce canopy throughfall and the runoff Mg flux (*p* > 0.05). The regionally valid causal relationship between bedrock dissolution and net export of Mg *via* runoff thus may not be relevant for UDL. In contrast to UDL, Mg export from completely forested LYS (mean of 1.9 kg ha^-1^ yr^-1^) was significantly higher than Mg throughfall flux (mean of 1.3 kg ha^-1^ yr^-1^; *p* < 0.05). Magnesium in spruce needles at LYS was isotopically different from Mg in open-area precipitation and throughfall, indicating Mg isotope fractionation associated with chlorophyll formation [34, 64], and possibly also lower rates of incorporation of deposited Mg into the needles, compared to UDL. Data from hydrochemical input*–*output monitoring are consistent with a significant geogenic contribution to Mg exported from LYS (*cf*., [41]). The discrepancy between the monitoring (net Mg export *via* runoff) and isotope data (different δ^26^Mg values of runoff and bedrock) at LYS remains unexplained at least until temporal variability in the δ^26^Mg values of atmospheric deposition and runoff is quantified.

Both bedrock types at the felsic sites LYS and UDL are Mg-poor (0.15–0.36 wt. % MgO; S1 Table). We suggest that a between-site comparison of Mg fluxes may provide an additional non-isotope insight into the origin of Mg in UDL runoff: While orthogneiss at UDL contains only about twice more Mg than leucogranite at LYS, the runoff Mg flux at UDL in 2013 was nine times higher than the runoff Mg flux at LYS. Over the 19-year monitoring period, the mean annual Mg export from UDL was five times higher than that from LYS. We suggest that the higher Mg export from UDL may be causally related to the higher atmospheric pollution level at this site. Input Mg flux *via* throughfall in 2013 was seven times higher at UDL than at LYS. Over the 19-year monitoring period, input Mg flux *via* throughfall was six times higher at UDL than that at LYS. The between-site comparison of non-isotope data is thus consistent with a relatively large contribution of present-day deposition to runoff Mg at UDL.

Based on temporal changes in the isotope composition of dissolved Mg at UDL, data in Fig 4 may be viewed as supporting evidence for an important role of atmospherically deposited Mg in runoff generation. Isotope composition of Mg in runoff followed the changes in δ^26^Mg values of atmospheric deposition (an increase from the dormant season to the end of growing season; Fig 4). The linkage between deposited Mg and exported Mg was mediated by soil waters (significant positive relationship between precipitation and soil water, *R* = 0.96). From a comparison of Fig 4 with Fig 3B it follows that base flow, sampled in mid-winter, may have contained a large proportion of bedrock- or dolomite-derived Mg even at UDL. The evidence for a major role of lithogenic Mg in winter-time UDL runoff, however, is inconclusive because also the δ^26^Mg value of deposition in winter was low. During the growing season, recent precipitation likely became the main control of the δ^26^Mg value of UDL runoff.

We note that some previous studies [37, 65] have observed seasonality in δ^26^Mg values of water samples in small catchments that was better documented than in our UDL study based only on one sample per season.

### Leaching of dolomite at UDL

There was a large difference between the δ^26^Mg value of the dolomite (-2.55 ‰) and that of UDL runoff (-1.52 ‰; Fig 3B). Magnesium isotope systematics indicated that 33 to six years after application of dolomite on the UDL surface Mg export *via* surface runoff was small to negligible. The mean δ^26^Mg values of bulk soil at individual soil depths were mostly higher than -2.00 ‰ (S2 Table), and were thus also different from the low δ^26^Mg signature of dolomite. Magnesium in bulk soil of UDL was isotopically nearly identical with xylem and may have been mainly controlled by recycled organic Mg (*cf*., [32]), along with high-δ^26^Mg products of isotope-selective weathering and soil exchange processes. The cumulative input of 1.2 tons of dolomite-Mg per ha represents mere 1.8 wt. % of exchangeable Mg present in the soil to the depth of 10 cm (forest floor and soil data from S1B Fig). If aboveground vegetation and roots are additionally included in the comparison, the total dolomite Mg corresponds to a mere 1.6 wt. % of the total Mg pool. Even if all Mg supplied to the UDL ecosystem by liming remained immobilized in the soil and biomass, its isotope composition would hardly affect the total Mg isotope signature of the topmost soil horizons.

## Conclusions

We combined long-term input–output Mg budgets with short-term Mg isotope systematics in an attempt to preliminarily apportion Mg sources for surface runoff in three small Central European catchments. Magnesium isotope ratios were determined in May 2012 at two sites (LYS and PLB), and in January, April, July and October 2013 at one site (UDL). Whereas the serpentinite catchment PLB mostly exported bedrock-derived Mg, the UDL catchment, underlain by orthogneiss, exported mainly atmospheric and organically-cycled Mg. During winter, the δ^26^Mg value of UDL runoff converged to the low δ^26^Mg value of bedrock, however, the δ^26^Mg value of atmospheric deposition also decreased, and therefore deposited Mg may have controlled the δ^26^Mg value of runoff even during the dormant season. Nine tons of dolomite per ha introduced to UDL to mitigate acid rain 33 to six years before the Mg isotope study did not significantly affect the Mg export *via* runoff. The third site, LYS, underlain by leucogranite, exhibited Mg isotope systematics similar to UDL, but both atmospheric deposition and runoff Mg fluxes at LYS were significantly lower than at UDL. Because the δ^26^Mg value of runoff at LYS was four times closer to the δ^26^Mg value of atmospheric deposition than to the δ^26^Mg value of bedrock, atmospheric Mg was likely a major contributor to runoff Mg. This finding from May 2012, however, is not in agreement with long-term input–output mass balances that indicated net export of Mg from the catchment. Similar δ^26^Mg values of LYS runoff and spruce biomass were consistent with a major proportion of biologically cycled dissolved Mg in runoff. High-δ^26^Mg magnesium in runoff from the two felsic catchments (LYS and UDL), relative to bedrock, indicated that solid weathering products (mainly Al-Fe sesquioxides) did not accumulate high-δ^26^Mg geogenic magnesium. This contrasts with previous studies reporting hydrological export of low-δ^26^Mg magnesium resulting from bedrock dissolution, and formation of high-δ^26^Mg clays. This discrepancy was likely caused by negligible rates of formation of secondary clay minerals at the high elevations of our study sites. The within-site variability in δ^26^Mg values was low at PLB, a site characterized by high Mg availability due to bedrock dissolution. The variability in δ^26^Mg values at PLB was dominated by mixing of geogenic with atmospheric Mg, with only a small contribution of biological Mg fractionations. In contrast, within-site isotope-selective processes served as important controls of δ^26^Mg variability in the studied felsic catchments LYS and UDL. Further studies will be needed to statistically evaluate the spatial and temporal δ^26^Mg variability within individual solid and liquid sample types.

## Supporting information

S1 Fig Schematic representation of the magnitude of Mg pool sizes and fluxes at the three study sites. Exchangeable Mg pool sizes are given for individual soil compartments, and total Mg pool sizes are given for all other sample types. δ^26^Mg marked with an asterisk were obtained in different soil depth intervals, compared to pool size measurements. Both Mg pool sizes and Mg fluxes are for the year 2015.
(TIF)

S1 Table Chemical analysis (wt. %) of the silicate bedrock at the study sites. (DOCX)

S2 Table Mg concentrations and Mg isotope composition of various types of samples. (DOCX)

S3 Table Statistical analysis of temporal changes in Mg isotope composition of water samples from UDL. Statistical significant correlations are in bold.
(DOCX)
