# Supplementary figures and images for: Controls on δ26Mg variability in three Central European headwater catchments characterized by contrasting bedrock chemistry and contrasting inputs of atmospheric pollutants
Source: PLoS One. 2020 Nov 30;15(11):e0242915. doi: 10.1371/journal.pone.0242915 (PMC7703950; doi:10.1371/journal.pone.0242915)

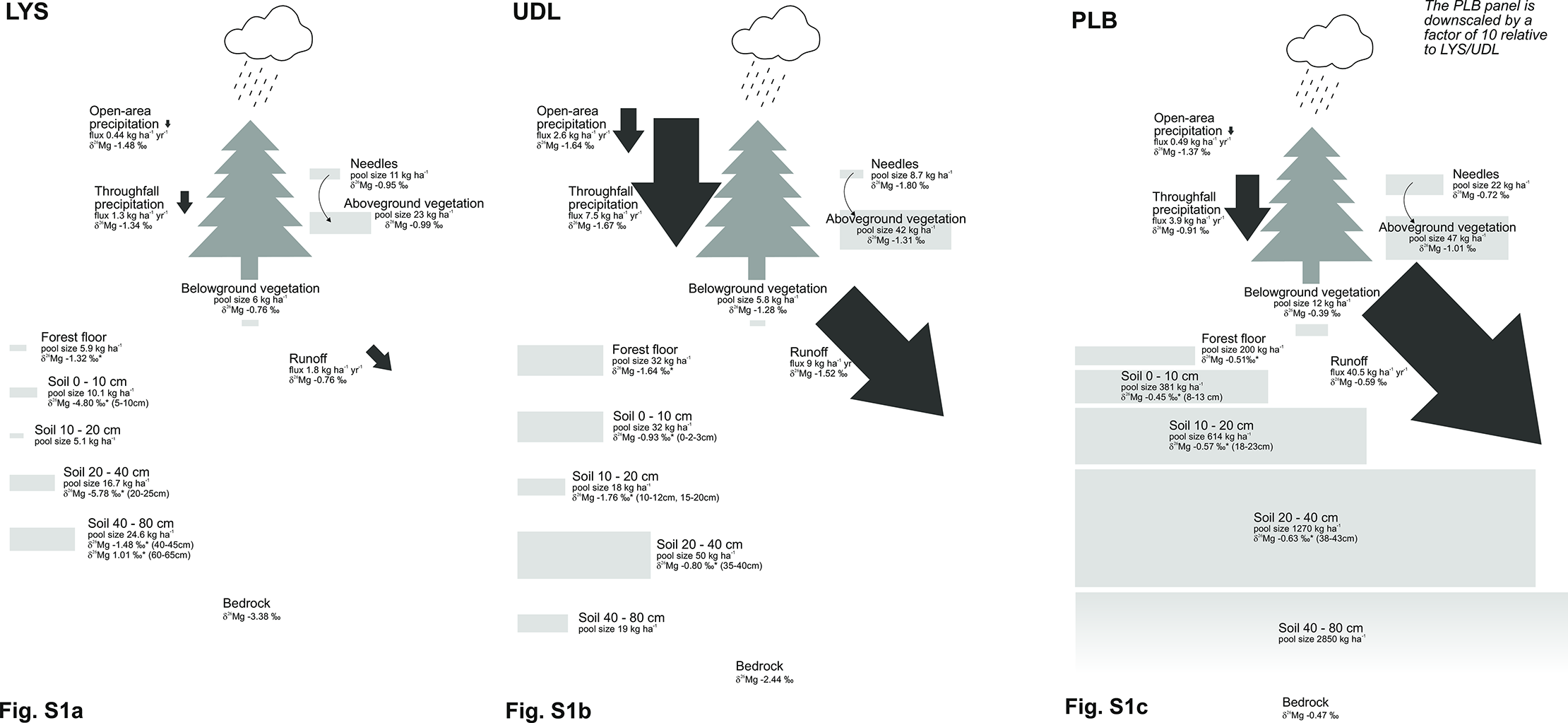

Supplement: S1 Fig — Exchangeable Mg pool sizes are given for individual soil compartments, and total Mg pool sizes are given for all other sample types. δ26Mg marked with an asterisk were obtained in different soil depth intervals, compared to pool size measurements. Both Mg pool sizes and Mg fluxes are for the year 2015. (TIF) [file pone.0242915.s001.tif]
