# Supplementary material for: Controls on δ26Mg variability in three Central European headwater catchments characterized by contrasting bedrock chemistry and contrasting inputs of atmospheric pollutants
Source: PLoS One. 2020 Nov 30;15(11):e0242915. doi: 10.1371/journal.pone.0242915 (PMC7703950; doi:10.1371/journal.pone.0242915)
Supplement: S1 Table — (DOCX) [file pone.0242915.s002.docx]

S1 Table. Chemical analysis (wt. %) of the silicate bedrock at the study sites.

| **Site**  **Bedrock** | **LYS**  granite | | **UDL**  gneiss | | **PLB**  serpentinite |
| --- | --- | --- | --- | --- | --- |
|  | Drilling  28m depth | Outcrop | Drilling  28m depth | Outcrop | Outcrop |
| SiO_2_ | 72.6 | 73 | 74.7 | 75.2 | 41 |
| Al_2_O_3_ | 13.7 | 14 | 12.7 | 12.8 | 1.2 |
| Fe_2_O_3_ | 0.32 | 0.7 | 1.64 | 0.49 | 6.4 |
| FeO | 0.72 | 1.4 | 0.33 | 1.35 | 1.5 |
| MnO | 0.05 | 0.07 | 0.03 | 0.04 | 0.13 |
| MgO | 0.15 | 0.1 | 0.36 | 0.16 | 36 |
| CaO | 0.43 | 0.5 | 0.40 | 0.32 | 0.3 |
| Na_2_O | 3.00 | 2.9 | 2.47 | 3.09 | 0.02 |
| K_2_O | 5.67 | 4.4 | 5.70 | 4.53 | 0.02 |
| P_2_O_5_ | 0.37 | 0.3 | 0.17 | 0.21 | 0.01 |
| H_2_O^+^ | 1.11 | 1.2 | 1.09 | 0.97 | 12 |
| H_2_O^-^ | 0.13 | 0.3 | 0.25 | 0.19 | 0.9 |
