# Supplementary material for: Controls on δ26Mg variability in three Central European headwater catchments characterized by contrasting bedrock chemistry and contrasting inputs of atmospheric pollutants
Source: PLoS One. 2020 Nov 30;15(11):e0242915. doi: 10.1371/journal.pone.0242915 (PMC7703950; doi:10.1371/journal.pone.0242915)
Supplement: S2 Table — (DOCX) [file pone.0242915.s003.docx]

S2 Table. Mg concentrations and Mg isotope composition of various types of samples.

| **Site** | **Sample type** | **Depth in soil (cm)** | **Sampling period** | **Mg concentration** | **δ^26^Mg relative to DSM (‰)** | **2 s.e.** |
| --- | --- | --- | --- | --- | --- | --- |
| LYS | Open-area precipitation |  | 30/4/2012 – 30/5/2012 | 0.04 mg L^-1^ | -1.48 | 0.07 |
|  | Spruce throughfall precipitation |  | 30/4/2012 – 30/5/2012 | 0.14 | -1.34 | 0.10 |
|  | Runoff |  | 30/5/2012 | 0.55 | -0.76 | 0.04 |
|  | Gravitational lysimeters | -10 | 20/3/2012 – 30/5/2012 | 0.12 | -1.49 | 0.11 |
|  | Gravitational lysimeters | -20 | 20/3/2012 – 30/5/2012 | 0.09 | -1.36 | 0.13 |
|  | Tension lysimeters | -60 | 20/3/2012 – 30/5/2012 | 0.07 | -1.26 | 0.11 |
|  | Tension lysimeters | -90 | 20/3/2012 – 30/5/2012 | 0.12 | -0.65 | 0.15 |
|  | Forest floor |  | November 2012 | 336 ppm | -1.32 | 0.11 |
|  | Soil | 5-10 | November 2012 | 37 | -4.80 | 0.03 |
|  | Soil | 20-25 | November 2012 | 8.79 | -5.78 | 0.11 |
|  | Soil | 40-45 | November 2012 | 5.38 | -1.48 | 0.13 |
|  | Soil | 60-65 | November 2012 | 5.38 | -1.01 | 0.10 |
|  | Spruce needles |  | November 2012 | 721 | -0.95 | 0.11 |
|  | Spruce bark |  | November 2012 | 456 | -0.82 | 0.05 |
|  | Fine roots (spruce) |  | November 2012 | 586 | -0.76 | 0.15 |
|  | Xylem |  | May 2016 | 353 | -0.99 | 0.04 |
|  | Whole rock |  | November 2012 | 645 | -3.38 | 0.05 |
| UDL | Open-area precipitation |  | January 2013 | 0.12 mg L^-1^ | -1.91 | 0.04 |
|  | Open-area precipitation |  | April 2013 | 0.34 | -1.74 | 0.08 |
|  | Open-area precipitation |  | July 2013 | 0.24 | -1.53 | 0.05 |
|  | Open-area precipitation |  | October 2013 | 0.10 | -1.38 | 0.13 |
|  | Spruce throughfall precipitation |  | January 2013 | 0.30 | -1.68 | 0.10 |
|  | Spruce throughfall precipitation |  | April 2013 | 1.40 | -1.78 | 0.17 |
|  | Spruce throughfall precipitation |  | July 2013 | 1.15 | -1.72 | 0.13 |
|  | Spruce throughfall precipitation |  | October 2013 | 0.81 | -1.51 | 0.08 |
|  | Runoff |  | January 2013 | 1.30 | -1.98 | 0.02 |
|  | Runoff |  | April 2013 | 0.71 | -1.59 | 0.08 |
|  | Runoff |  | July 2013 | 0.76 | -1.26 | 0.07 |
|  | Runoff |  | October 2013 | 0.61 | -1.26 | 0.08 |
|  | Tension lysimeters | -30 | January 2013 | 0.66 | -2.04 | 0.08 |
|  | Tension lysimeters | -50 | January 2013 | 0.63 | -2.13 | 0.17 |
|  | Tension lysimeters | -30 | April 2013 | 1.01 | -2.03 | 0.09 |
|  | Tension lysimeters | -50 | April 2013 | 0.88 | -1.87 | 0.05 |
|  | Tension lysimeters | -30 | July 2013 | n.d. | -1.74 | 0.07 |
|  | Tension lysimeters | -50 | July 2013 | 0.58 | -1.91 | 0.06 |
|  | Tension lysimeters | -30 | October 2013 | 0.48 | -1.64 | 0.12 |
|  | Tension lysimeters | -50 | October 2013 | 0.57 | -1.85 | 0.09 |
|  | Forest floor |  | November 2012 | 713 ppm | -1.64 | 0.09 |
|  | Soil  (*low elevation*) | 0-3 | November 2012 | 81.1 | -0.46 | 0.17 |
|  | Soil  (*low elevation*) | 10-12 | November 2012 | 37.6 | -2.09 | 0.11 |
|  | Soil  (*low elevation*) | 35-40 | November 2012 | 39.2 | -1.38 | 0.10 |
|  |  |  |  |  |  |  |
|  | Soil  (*high elevation*) | 0-2 | November 2012 | n.d. | -1.40 | 0.02 |
|  | Soil  (*high elevation*) | 15-20 | November 2012 | n.d. | -1.44 | 0.03 |
|  | Soil  (*high elevation*) | 35-40 | November 2012 | n.d. | -0.22 | 0.15 |
|  | Spruce needles |  | November 2012 | 868 | -1.80 | 0.09 |
|  | Spruce bark |  | November 2012 | 984 | -1.34 | 0.01 |
|  | Fine roots (spruce) |  | November 2012 | 705 | -1.28 | 0.13 |
|  | Xylem |  | May 2016 | 248 | -1.31 | 0.04 |
|  | Whole rock  (*low elevation*) I |  | November 2012 | 1790 | -2.26 | 0.01 |
|  | Whole rock  (*high elevation*) II |  | November 2012 | 1790 | -2.62 | 0.08 |
| PLB | Open-area precipitation |  | 30/4/2012 -30/5/2012 | 0.06 mg L^-1^ | -1.37 | 0.15 |
|  | Spruce throughfall precipitation |  | 30/4/2012 -30/5/2012 | 1.12 | -0.91 | 0.11 |
|  | Runoff |  | 30/5/2012 | 27.7 | -0.59 | 0.08 |
|  | Gravitational lysimeters | -10 | 20/3/2012 – 30/5/2012 | 6.30 | -0.35 | 0.05 |
|  | Gravitational lysimeters | -20 | 20/3/2012 – 30/5/2012 | 7.44 | -0.40 | 0.09 |
|  | Tension lysimeters | -60 | 20/3/2012 – 30/5/2012 | 9.43 | -0.56 | 0.16 |
|  | Tension lysimeters | -90 | 20/3/2012 – 30/5/2012 | 17.6 | -0.79 | 0.09 |
|  | Forest floor |  | November 2012 | 336 ppm | -0.51 | 0.03 |
|  | Soil | 8-13 | November 2012 | 952 | -0.45 | 0.03 |
|  | Soil | 18-23 | November 2012 | 574 | -0.57 | 0.07 |
|  | Soil | 38-43 | November 2012 | 1070 | -0.63 | 0.03 |
|  | Spruce needles |  | November 2012 | 1520 | -0.72 | 0.06 |
|  | Spruce bark |  | November 2012 | 589 | -0.45 | 0.05 |
|  | Fine roots (spruce) |  | November 2012 | 2190 | -0.39 | 0.09 |
|  | Xylem |  | May 2016 | 305 | -1.01 | 0.04 |
|  | Whole rock |  | November 2012 | 217000 | -0.47 | 0.04 |
|  |  |  |  |  |  |  |
