# Supplementary material for: Controls on δ26Mg variability in three Central European headwater catchments characterized by contrasting bedrock chemistry and contrasting inputs of atmospheric pollutants
Source: PLoS One. 2020 Nov 30;15(11):e0242915. doi: 10.1371/journal.pone.0242915 (PMC7703950; doi:10.1371/journal.pone.0242915)
Supplement: S3 Table — Statistical significant correlations are in bold. (DOCX) [file pone.0242915.s004.docx]

S3 Table. Statistical analysis of temporal changes in Mg isotope composition of water samples from UDL.

| Sample types | Correlation | 95% Confidence interval | P-value |
| --- | --- | --- | --- |
| Soilwater 30 cm – Open-area precipitation | **0.96** | **(0.34, 1.00)** | **0.018** |
| Soilwater 50 cm – Open-area precipitation | 0.78 | (-0.54, 1.00) | 0.112 |
| Runoff – Open-area precipitation | **0.95** | **(0.20, 1.00)** | **0.024** |
| Throughfall – Open-area precipitation | 0.62 | (-0.73, 1.00) | 0.190 |
| Soilwater 50 cm – Soilwater 30 cm | 0.58 | (-0.75, 1.00) | 0.209 |
| Runoff – Soilwater 30 cm | 0.88 | (-0.27, 1.00) | 0.061 |
| Throughfall – Soilwater 30 cm | 0.71 | (-0.64, 1.00) | 0.144 |
| Runoff – Soilwater 50 cm | 0.86 | (-0.35, 1.00) | 0.072 |
| Throughfall – Soilwater 50 cm | 0.14 | (-0.90, 1.00) | 0.428 |
| Throughfall - Runoff | 0.35 | (-0.86, 1.00) | 0.325 |
